# The Effect of Coronary Artery Disease on the Prognosis of Hypertrophic Cardiomyopathy: A Multi-Center Cohort Study

**DOI:** 10.31083/RCM25045

**Published:** 2025-01-16

**Authors:** Guoqing Hou, Qian Liao, Huihui Ma, Yan Shu, Shengzhi Zeng, Yongmei Zhou, Liangjun Luo, Gang Zhao, Tao He, Mingjiang Liu, Jianhong Tao, Wei Hua, Xiaoping Li

**Affiliations:** ^1^Department of Cardiology, Affiliated Hospital of Southwest Medical University, 646000 Luzhou, Sichuan, China; ^2^Department of Cardiology, Chinese Academy of Sciences Sichuan Translational Medicine Research Hospital, 610072 Chengdu, Sichuan, China; ^3^Department of Cardiology, Sichuan Provincial People's Hospital, University of Electronic Science and Technology of China, 610072 Chengdu, Sichuan, China; ^4^Department of Cardiology, Guanghan People's Hospital, 618300 Guanghan, Sichuan, China; ^5^Clinical Lab, The First People's Hospital of Liangshan Yi Autonomous Prefecture, 615000 Xichang, Sichuan, China; ^6^Department of Cardiology, The First People's Hospital of Liangshan Yi Autonomous Prefecture, 615000 Xichang, Sichuan, China; ^7^Department of Clinical Electrophysiology, Fuwai Hospital and Cardiovascular Institute, Chinese Academy of Medical Science and Peking Union Medical College, 100037 Beijing, China

**Keywords:** cardiomyopathy, hypertrophic, coronary artery disease, prognosis, sudden cardiac death

## Abstract

**Background:**

There is a shortage of patients with hypertrophic cardiomyopathy (HCM) with concurrent coronary artery disease (CAD), and the influence of CAD on the prognosis of patients with HCM is uncertain. This real-world cohort study was conducted to evaluate the prognosis of patients with patients with CAD.

**Methods:**

This cohort study of patients with HCM was conducted from May 2003 to September 2021. The total number of patients enrolled was 2167, and the mean follow-up period was 6.4 years (interquartile range 2.8–9.5 years). Sudden cardiac death (SCD), cardiovascular death, and all-cause mortality were assessed as outcomes. Using logistic regression, nine indicators were selected for 1:1 propensity score matching (PSM). Additionally, Kaplan–Meier survival curves and Cox proportional hazards regression analyses were used to assess the impact of CAD on the prognosis of patients with HCM.

**Results:**

During an average of 6.4 years of follow-up, of the 2167 patients enrolled, 446 (20.6%) died. The patients were classified into two groups: CAD (n = 480) and non-CAD (n = 1,687). After imputation of missing values using the mean and 1:1 propensity score matching, there was no difference in SCD (log-rank χ^2^ = 0.4, *p* = 0.540), cardiovascular death (log-rank χ^2^ = 0.1, *p* = 0.995) and all-cause mortality (log-rank χ^2^ = 0.1, *p* = 0.776) between the CAD and non-CAD groups. After imputation of missing values using the median and 1:1 propensity score matching, patients with and without CAD were not significantly different in terms of SCD (log-rank χ^2^ = 0.1, *p* = 0.948), cardiovascular death (log-rank χ^2^ = 0.1, *p* = 0.811), and all-cause mortality (log-rank χ^2^ = 0.5, *p* = 0.499). In the Cox analysis, CAD was not a significant independent predictor of SCD, cardiovascular death, or all-cause mortality in patients with HCM.

**Conclusions:**

In this study, it was observed that there was no statistically significant disparity in mortality rates between patients diagnosed with HCM who concurrently had CAD and those who did not exhibit CAD. This finding underscores the notion that the presence of CAD did not exert a notable influence on the incidence of SCD, cardiovascular death, or all-cause mortality, thereby emphasizing the complexity and multifaceted nature of mortality risk factors in HCM patients.

## 1. Introduction

Hypertrophic cardiomyopathy (HCM) is an endogenous myocardial heart disease marked by left ventricular hypertrophy without ventricular dilatation, affecting 1 in 500 people [1]. Although HCM is the most prevalent cause of sudden cardiac death (SCD) among young individuals, especially athletes, the annual rate of SCD in adults with HCM is only around 1% [2, 3]. In accordance with the European Society of Cardiology SCD risk prediction model and the 2020 American Heart Association/American College of Cardiology Foundation guideline, a number of factors are recognized as well-known clinical risk factors for SCD, including syncope, age, left atrial diameter, non-sustained ventricular tachycardia, left ventricular outflow tract obstruction, maximum left ventricular wall thickness, and family history of SCD at a young age [4, 5, 6]. Patients with HCM may develop coronary artery disease (CAD), and the proportion of patients with HCM with concurrent CAD ranges from 10% to 53% according to previous studies [7, 8, 9]. However, longitudinal evidence about the clinical implications of CAD in patients with HCM is limited [10, 11]. Moreover, whether CAD affects the outcomes of HCM is uncertain. Therefore, the aim of this study was to evaluate the long-term clinical outcomes of patients with HCM with concurrent CAD.

## 2. Methods

### 2.1 Study Population

**Fig. 1.**
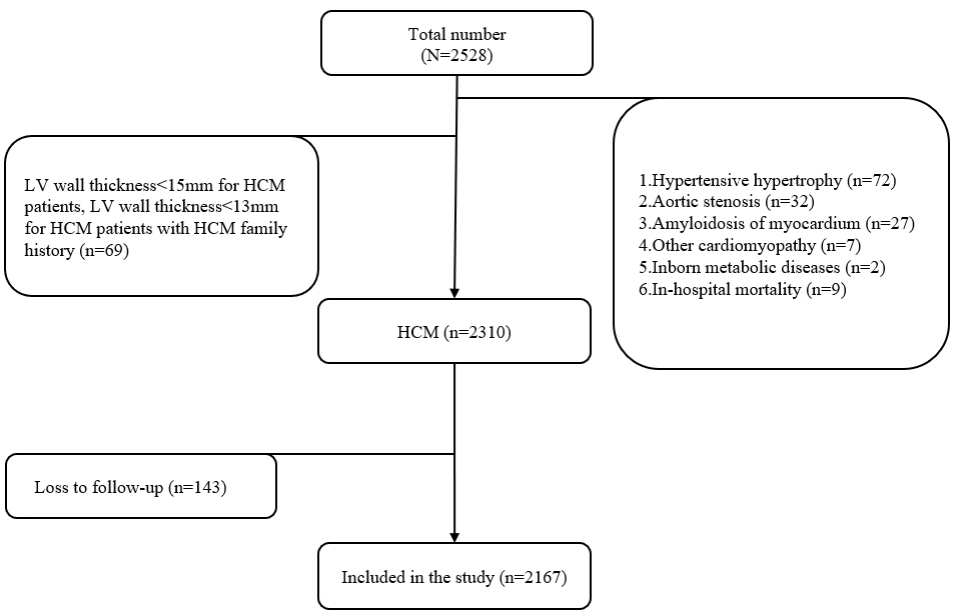
**Flowchart for patients involved in present study**. LV, left ventricular; HCM, hypertrophic cardiomyopathy.

Between May 2003 and September 2021, patients with HCM at 13 hospitals in China were enrolled. We collected patients’ general information, admission conditions, examination reports (including electrocardiogram, echocardiogram, chest X-ray, coronary computed tomography (CT), coronary angiography, etc.), laboratory test reports (such as myocardial markers, N-terminal pro-brain natriuretic peptide [NT-proBNP], liver and kidney function, blood routine, etc.), medication information, and other relevant data. Patients with hereditary metabolic illness that would lead to HCM were excluded. Patients who were unreachable within 6 months after discharge and had no police station record of death were considered lost cases and were excluded from the study cohort. Overall, 2167 patients were eventually enrolled for analysis (Fig. 1).

### 2.2 Diagnostic Criteria

HCM was diagnosed based on echocardiographic characteristics of ventricular myocardial hypertrophy. HCM was defined as a maximum wall thickness of ≥15 mm in the general population or ≥13 mm among those with a family history of HCM in adults. In children, HCM was diagnosed if the left ventricular wall thickness was more than two standard deviations greater than the predicted mean. Patients with cardiac or systemic diseases that lead to similar magnitudes of hypertrophy were excluded, such as cardiac amyloidosis, Noonan syndrome and Fabry disease [2, 4, 12]. The presence of atherosclerotic CAD was determined by coronary angiography or coronary CT angiography. For the left main coronary artery, a luminal diameter stenosis of >50% was considered as CAD, while for other major epicardial branches, stenosis of >70% was considered as CAD [10, 13]. In addition, patients who had a history of percutaneous coronary intervention, acute myocardial infarction and coronary artery bypass grafting surgery were also defined as having CAD [11]. Patients who had not undergone coronary angiography or coronary CT and this could not be definitively diagnosed with CAD were excluded.

### 2.3 Follow-up and Endpoints

The interval between the first assessment and the final contact date or the moment of death was referred to as the follow-up period. All-cause mortality was the primary endpoint. Cardiovascular mortality and SCD were the secondary endpoints. The incidences of death from cardiac transplantation, heart failure, embolism, cerebrovascular disease, and stroke were included in the endpoint of cardiovascular death. Individuals who had previously been in a stable clinical state and died suddenly or collapsed unexpectedly within 24 hours of not being seen were said to have suffered from SCD. Patients’ medical records were reviewed, including those related to telephone interviews, clinic and hospital attendance, and information about survival status received from the local police department and the Centers for Disease Control and Prevention, and data on the incidence of SCD, cardiovascular death, and all-cause mortality at follow-up were collected.

### 2.4 Data Analysis

The biochemical indicators were measured by the Roche Cobas c 701 Analyzer (Roche Diagnostics GmbH, 882 Ichige, Hitachinaka-shi, Ibaraki-ken, 312-8504, Japan). The data were analyzed using SPSS 26.0. Categorical variables are presented as numbers with percentages, while continuous variables are presented as the mean ± standard deviation or median (interquartile range). Normally distributed continuous variables were compared using the Student’s *t*-test; otherwise, Wilcoxon’s rank-sum test was used. Fisher’s exact test or the χ^2^ test was used to evaluate associations in contingency tables. By calculating the mean of the non-missing values and assigning this mean value to the missing values, the missing values are effectively imputed. By conducting a sensitivity analysis that compares different methods of imputing missing values (mean imputation vs. median imputation), we aim to strengthen the robustness and reliability of the findings presented in this paper. The Kaplan–Meier method was adopted to calculate survival with 95% confidence intervals (CI).

Using univariate logistic regression, we carefully selected the indicators for propensity score matching (PSM), ultimately identifying nine variables with *p* < 0.1 including age, hypertension, diabetes mellitus, tobacco use, family history of HCM, total bilirubin, direct bilirubin, high-density lipoprotein, and low-density lipoprotein. Then, the nine indicators were subjected to caliper matching, incorporated into a 1:1 PSM process, ultimately establishing a matched cohort of 404 HCM patients with CAD and 404 HCM patients without CAD. This rigorous matching strategy enabled us to create comparable groups for subsequent analysis, enhancing the accuracy and reliability of our findings. Variables with *p* < 0.05 at baseline combined with clinical experience were included in the univariate Cox regression analysis. Similarly, variables with *p* < 0.05 in the univariate Cox regression analysis were included in the multivariate regression analysis. The multivariate Cox proportional hazards regression model used a stepwise variable selection procedure. Statistics were judged significant at *p* < 0.05.

## 3. Results

### 3.1 Baseline Characteristics

Overall, 2167 consecutive patients with HCM were included in the analysis. The median age was 55 ± 15 years, with 1372 (63.3%) being male, 190 (8.8%) having a family history of HCM, and 55 (2.5%) having an implanted cardioverter defibrillator (ICD). CAD was observed in 480 patients (22.2%), while 1687 (77.8%) did not have CAD. Table 1 shows the baseline clinical characteristics of the two groups. In the unmatched cohort, patients with CAD had higher systolic and diastolic blood pressures, shorter QRS and QTc durations, older age, and more frequent histories of hypertension and diabetes mellitus than patients with HCM without CAD. Patients with HCM and CAD more commonly had a history of smoking (42.9% vs. 35.0%), and the incidence of a family history of HCM was lower in those with CAD (4.2% vs. 10.1%). There were no significant differences between the two groups in terms of New York Heart Association functional class III/IV, PR duration, QT duration, left ventricular (LV) diameters, left atrial diameters (LA), right ventricular (RV) diameters , interventricular septum (IVS), left ventricular ejection fraction (LVEF), NT-proBNP, ICD, syncope, atrial fibrillation (AF), and other medical histories. In the matched cohort, patients with HCM with CAD had shorter QT and corrected QT interval (QTc) durations than those without CAD. There were no statistically significant differences for any of the other indicators in the matched cohort.

**Table 1.** **Baseline patient characteristics**.

Value		Unmatched (n = 2167)			Missing (%)	Matched (n = 808)		
		CAD (n = 480)	No CAD (n = 1687)	*p* value		CAD (n = 404)	No CAD (n = 404)	*p* value
Age, years		60 ± 13	53 ± 15	<0.001*	0.00	59 ± 13	59 ± 14	1.000#
Male, n (%)		308 (64.2)	1064 (63.1)	0.660	0.00	259 (64.1)	253 (62.6)	0.661
History								
	Syncope, n (%)	54 (11.3)	241 (14.3)	0.087	0.00	45 (11.1)	50 (12.4)	0.585
	NSVT, n (%)	36 (7.8)	109 (6.7)	0.380	3.37	30 (7.4)	25 (6.2)	0.485
	HOCM, n (%)	202 (42.1)	791 (46.9)	0.062	0.00	170 (42.1)	174 (43.1)	0.776
	AF, n (%)	86 (17.9)	294 (17.4)	0.804	0.00	74 (18.3)	87 (21.5)	0.252
	Hypertension, n (%)	294 (61.3)	632 (37.5)	<0.001*	0.00	223 (55.2)	223 (55.2)	1.000#
	Hyperlipidemia, n (%)	146 (30.4)	373 (22.1)	<0.001*	0.00	117 (29.0)	98 (24.3)	0.130
	DM, n (%)	90 (18.8)	156 (9.2)	<0.001*	0.00	57 (14.1)	54 (13.4)	0.759#
	ICD, n (%)	8 (1.7)	47 (2.8)	0.168	0.00	7 (1.7)	8 (2.0)	0.794
	Tobacco use, n (%)	206 (42.9)	590 (35.0)	0.001*	0.00	168 (41.6)	162 (40.1)	0.668#
	FHCM, n (%)	20 (4.2)	170 (10.1)	<0.001*	0.00	20 (5.0)	12 (3.0)	0.149#
	NYHA III/IV, n (%)	205 (42.7)	661 (39.2)	0.164	0.00	170 (42.1)	165 (40.8)	0.721
Admission vital sign								
	Heart rate, beat/min	74 ± 17	75 ± 17	0.666	0.00	74 ± 17	74 ± 16	0.872
	SBP, mmHg	130 ± 23	125 ± 23	<0.001*	0.00	128 ± 22	128 ± 23	0.968
	DBP, mmHg	77 ± 13	76 ± 14	0.017*	0.00	77 ± 13	78 ± 14	0.464
Laboratory values at admission								
	AST, IU/L	31.2 ± 25.3	32.8 ± 26.8	0.621	8.44	31.7 ± 13.5	28.0 ± 15.5	0.050
	ALT, IU/L	31.23 ± 22.9	32.6 ± 25.4	0.601	8.77	31.5 ± 29.8	28.2 ± 21.3	0.138
	TB, mmol/L	16.2 ± 7.8	17.0 ± 12.5	0.071	8.49	16.2 ± 7.5	16.1 ± 7.1	0.877#
	DB, mmol/L	4.0 ± 2.6	4.6 ± 2.6	0.009*	8.49	4.0 ± 2.6	4.0 ± 2.4	0.712#
	Glucose, mmol/L	6.1 ± 2.4	5.8 ± 2.7	0.025*	8.53	6.0 ± 2.2	5.9 ± 2.2	0.255
	CR, mmol/L	89.4 ± 58.7	85.0 ± 64.1	0.190	6.13	88.1 ± 59.6	90.1 ± 74.0	0.672
	TG, mmol/L	1.7 ± 1.2	1.6 ± 1.1	0.457	13.52	1.6 ± 1.0	1.6 ± 0.9	0.854
	CHO, mmol/L	4.4 ± 1.1	4.4 ± 1.1	0.316	13.61	4.4 ± 1.0	4.4 ± 1.1	0.834
	HDL, mmol/L	1.1 ± 0.3	1.2 ± 0.4	0.134	17.02	1.2 ± 0.3	1.2 ± 0.3	0.445#
	LDL, mmol/L	2.5 ± 0.8	2.6 ± 0.9	0.057	16.84	2.5 ± 0.8	2.5 ± 0.8	0.933#
	Log NT-proBNP	3.1 ± 0.5	3.1 ± 0.5	0.722	28.61	3.1 ± 0.4	3.1 ± 0.4	0.777
Electrograph data								
	QRS, ms	104.0 ± 24.0	107.9 ± 28.6	0.011*	12.00	103.8 ± 21.7	106.2 ± 25.2	0.143
	QT, ms	414.9 ± 50.6	420.4 ± 52.2	0.055	12.41	414.3 ± 48.6	422.2 ± 46.3	0.019*
	QTc, ms	450.5 ± 52.5	457.4 ± 48.4	0.011*	13.15	450.6 ± 46.9	457.6 ± 42.9	0.027*
	PR, ms	172.2 ± 39.4	169.6 ± 36.3	0.232	19.80	171.3 ± 35.5	172.1 ± 29.0	0.727
Echocardiography data								
	LVd, mm	44.2 ± 6.9	44.3 ± 6.0	0.740	8.35	44.2 ± 5.9	44.4 ± 6.8	0.596
	LA, mm	39.8 ± 6.9	40.0 ± 7.2	0.584	7.61	39.6 ± 6.9	40.3 ± 7.1	0.208
	RV, mm	20.0 ± 3.2	19.9 ± 3.3	0.618	13.29	19.9 ± 3.0	19.7 ± 2.9	0.214
	IVS, mm	18.1 ± 4.7	18.4 ± 5.3	0.266	6.88	18.1 ± 4.6	17.8 ± 4.9	0.533
	LVEF, %	66.9 ± 8.9	66.1 ± 9.6	0.100	9.18	66.5 ± 8.7	65.9 ± 8.6	0.356
Medications at admission								
	Aldosterone antagonist, n (%)	88 (18.3)	320 (19.0)	0.724	0.32	73 (18.1)	78 (19.3)	0.652
	β-Antagonists, n (%)	386 (80.4)	1310 (78.0)	0.251	0.32	321 (79.5)	308 (76.2)	0.271
	CCB, n (%)	132 (27.6)	390 (23.3)	0.052	0.69	108 (26.7)	101 (25.0)	0.574
	Diuretic, n (%)	155 (32.3)	518 (30.8)	0.528	0.18	130 (32.2)	127 (31.4)	0.821

**Notes**: Data are expressed as the mean ± SD, medians, or as percentages. *p* value was obtained from independent-sample *t*-test. “*” represents *p* < 0.05, indicating statistical significance. “#” represents the indicators selected through logistic regression for use in propensity score matching. **Abbreviations**: NSVT, non-sustained ventricular tachycardia; AF, atrial fibrillation; DM, diabetes mellitus; ICD, implantable cardioverter defibrillator; FHCM, family history of HCM; NYHA, New York Heart Association; SBP, systolic blood pressure; DBP, diastolic blood pressure; AST, aspartate aminotransferase; ALT, alanine aminotransferase; TB, total bilirubin; DB, direct bilirubin; CR, creatinine; TG, triglyceride; CHO, cholesterol; HDL, high-density lipoprotein; LDL, low-density lipoprotein; LVd, left ventricular diameter; LA, left atrium; RV, right ventricle; IVS, interventricular septum thickness; LVEF, left ventricular ejection fraction; CCB, calcium channel blockers; CAD, coronary artery disease; HOCM, hypertrophic obstructive cardiomyopathy; NT-proBNP,N-terminal pro-B-type natriuretic peptide; HCM, hypertrophic cardiomyopathy; QTc, corrected QT interval.

### 3.2 Follow-up Data

**Fig. 2.**
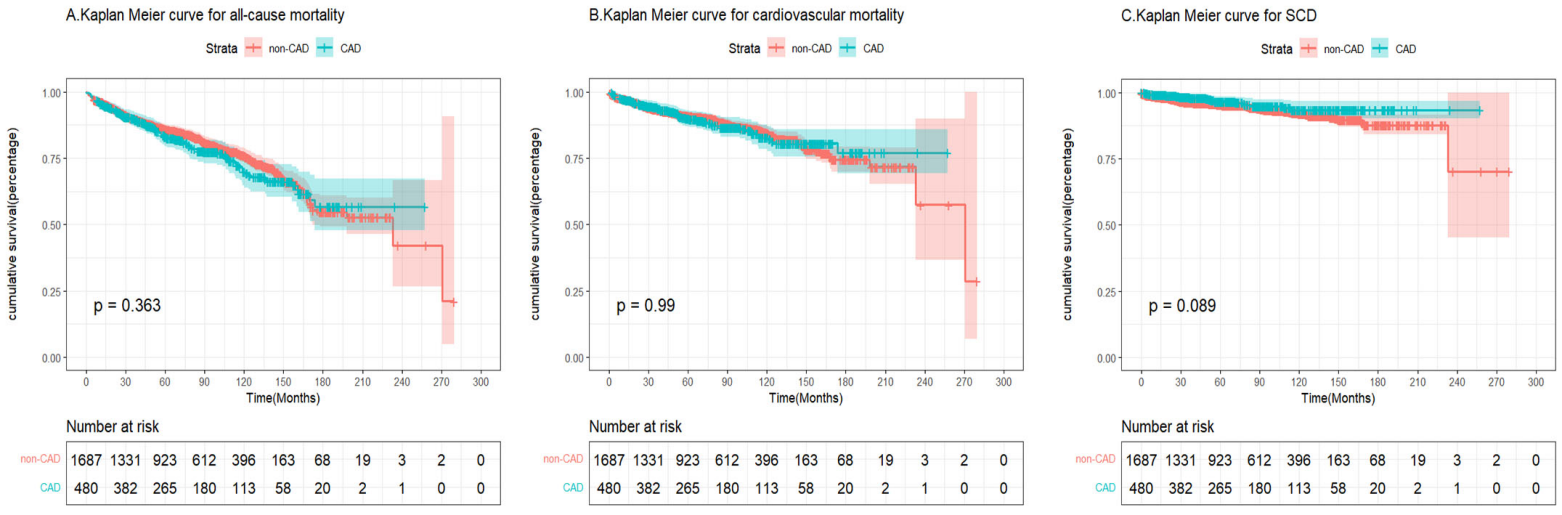
**Kaplan-Meier curves demonstrating the difference in all-cause mortality, cardiovascular mortality, and sudden cardiac death between coronary artery disease (CAD) and no CAD patients with HCM before matching**. (A) Kaplan Meier curve for all-cause mortality between CAD and no CAD patients with HCM before matching. (B) Kaplan Meier curve for cardiovascular mortality between CAD and no CAD patients with HCM before matching. (C) Kaplan Meier curve for SCD between CAD and no CAD patients with HCM before matching. HCM, hypertrophic cardiomyopathy; SCD, sudden cardiac death.

**Fig. 3.**
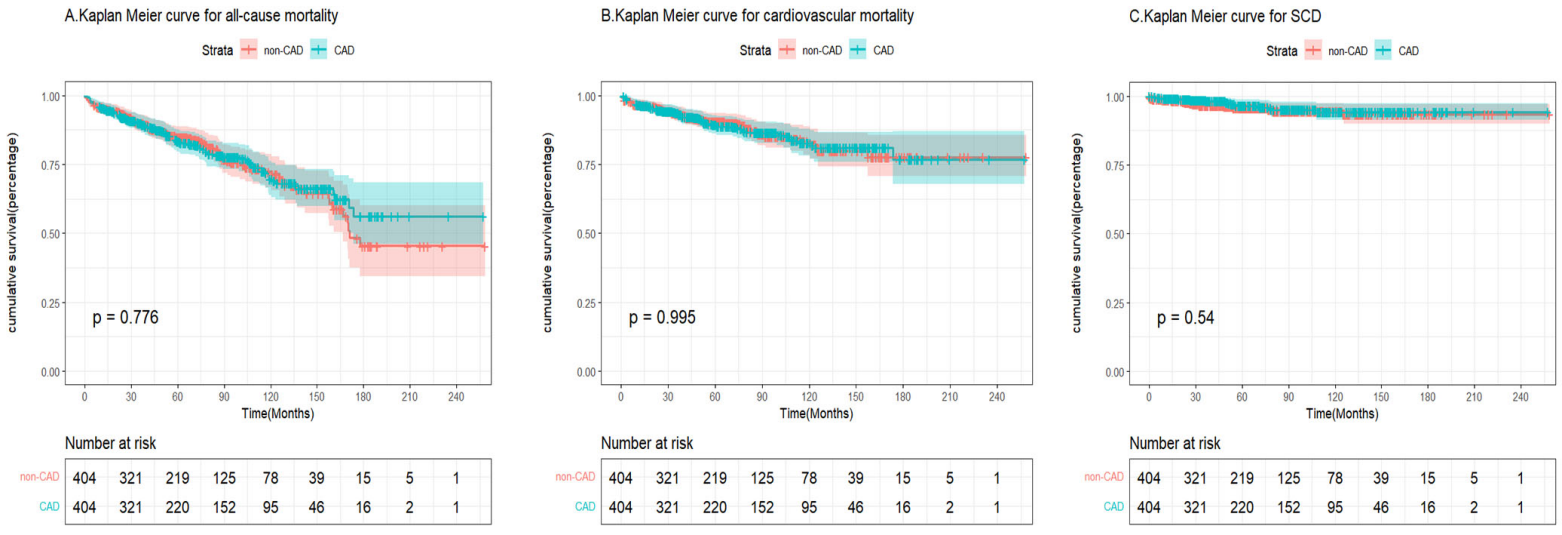
**Kaplan-Meier curves demonstrating the difference in all-cause mortality, cardiovascular mortality, and sudden cardiac death between CAD and no CAD patients with HCM after matching**. (A) Kaplan Meier curve for all-cause mortality between CAD and no CAD patients with HCM after matching. (B) Kaplan Meier curve for cardiovascular mortality between CAD and no CAD patients with HCM after matching. (C) Kaplan Meier curve for sudden cardiac death (SCD) between CAD and no CAD patients with HCM after matching. HCM, hypertrophic cardiomyopathy; CAD, coronary artery disease.

The mean duration of follow-up was 6.4 years (interquartile range 2.8–9.5 years), and 446 patients (20.6%) reached the primary endpoint, including three patients with cardiac transplantation. There were 260 cardiovascular deaths overall, 114 of which were due to SCD, 27 due to heart failure, 36 due to stroke, 3 due to cardiac transplantation, 2 due to myocardial infarction, and 78 due to other cardiac reasons (Table 2). Patients with HCM and CAD had a greater all-cause mortality rate (22.5%) than those without CAD (20.0%) (log-rank χ^2^ = 0.8, *p* = 0.363) (Table 2). In the unmatched cohort, patients with and without CAD were not significantly different in terms of SCD (log-rank χ^2^ = 2.9, *p* = 0.089), cardiovascular death (log-rank χ^2^ = 0.1, *p* = 0.990), and all-cause mortality (log-rank χ^2^ = 0.8, *p *= 0.363) (Fig. 2A–C). In the matched cohort, patients with and without CAD were not significantly different in terms of SCD (log-rank χ^2^ = 0.4, *p* = 0.540), cardiovascular death (log-rank χ^2^ = 0.1, *p* = 0.995), and all-cause mortality (log-rank χ^2^ = 0.1, *p* = 0.776) (Fig. 3A–C).

**Table 2.** **Cause of death according to absence or presence of CAD**.

	Unmatched (n = 2167)		Matched (n = 808)	
	No CAD (n = 1687)	CAD (n = 480)	No CAD (n = 404)	CAD (n = 404)
Death to any cause, n (%)	338 (20.0)	108 (22.5)	89 (22.0)	90 (22.3)
Cardiovascular death, n (%)	202 (20.0)	58 (12.1)	48 (11.9)	50 (12.4)
Sudden death, n (%)	96 (5.7)	18 (3.8)	17 (4.2)	14 (3.5)
Myocardial infarction, n (%)	0 (0.0)	2 (0.4)	0 (0.0)	1 (0.2)
Cardiac transplantation, n (%)	3 (0.2)	0 (0.0)	1 (0.2)	0 (0.0)
Heart failure, n (%)	19 (1.1)	8 (1.6)	4 (1.0)	8 (2.0)
Stroke, n (%)	31 (1.8)	5 (1.0)	15 (3.7)	5 (1.2)
^#^Others, n (%)	53 (3.1)	25 (5.2)	11 (2.7)	22 (5.4)
Non-cardiovascular death, n (%)	69 (4.1)	22 (4.6)	19 (4.7)	14 (3.5)
Unknown cause, n (%)	67 (4.0)	28 (5.8)	22 (5.4)	26 (6.4)

Note: ^#^Others refers to embolism, cerebrovascular disease, and other fatal heart diseases. CAD, coronary artery disease.

### 3.3 Univariate Cox and Multivariate Cox Proportional Hazards Regression Analyses

In the matched cohort, according to the univariate Cox proportional hazards regression analysis, age, sex, AF, hyperlipidemia, smoking history, heart rate, aspartate transaminase, direct bilirubin, creatinine, high-density lipoprotein, NT-proBNP, QT duration, left atrial diameter, and LVEF were significant predictors of death from any causes in patients with HCM patients when the clinical, laboratory, electrographic, and electrocardiographic data were considered. After adjusting for confounding variables, the analysis of all-cause mortality from the multivariate Cox proportional hazards regression analysis reveals that age, direct bilirubin, creatinine, NT-proBNP, and LVEF were strong independent predictors of all-cause mortality. However, survival did not differ significantly between the CAD and non-CAD groups (hazard ratio [HR] 0.958, 95% CI 0.715–1.285, *p* = 0.776) (Table 3).

**Table 3.** **Cox regression of all-cause mortality in patients with HCM**.

Variables	Univariate analysis		Multivariate analysis	
	HR (95% CI)	*p* value	HR (95% CI)	*p* value
CAD	0.958 (0.715–1.285)	0.776		
Age	1.050 (1.037–1.064)	<0.001*	1.037 (1.023–1.051)	<0.001*
Male	1.598 (1.184–2.158)	0.002*		
HOCM	0.781 (0.579–1.053)	0.105		
AF	1.912 (1.371–2.667)	<0.001*		
Hyperlipemia	0.517 (0.353–0.758)	<0.001*		
Tobacco use	0.728 (0.539–0.984)	0.039*		
Heart rate	1.015 (1.007–1.023)	<0.001*		
AST	1.007 (1.003–1.010)	<0.001*		
DB	1.127 (1.081–1.175)	<0.001*	1.088 (1.030–1.150)	0.003*
CR	1.002 (1.001–1.003)	<0.001*	1.003 (1.001–1.004)	0.002*
HDL	1.606 (1.020–2.531)	0.041*		
Log NT-proBNP	5.158 (3.756–7.085)	<0.001*	2.964 (2.015–4.360)	<0.001*
QT	0.997 (0.994–0.999)	0.037*		
IVS	1.022 (0.993–1.052)	0.134		
LA	1.024 (1.002–1.046)	0.030*		
LVEF	0.960 (0.944–0.976)	<0.001*	0.970 (0.953–0.987)	0.001*

**Notes: **“*” represents *p* < 0.05, indicating statistical significance. HCM, hypertrophic cardiomyopathy; HR, hazard ratio; 95% CI, 95% confidence interval; CAD, coronary artery disease; HOCM, hypertrophic obstructive cardiomyopathy; AF, atrial fibrillation; AST, aspartate aminotransferase; DB, direct bilirubin; CR, creatinine; HDL, high-density lipoprotein; NT-proBNP, N-terminal pro-B-type natriuretic peptide; IVS, interventricular septum; LA, left atrium; LVEF, left ventricular ejection fraction.

After adjusting for other related variables, the multivariate analysis revealed that age, creatinine (CR), NT-proBNP, QT duration, and LVEF were independent predictors of cardiovascular death (Table 4). In addition, after adjusting for other related variables, only CR was an independent predictor of SCD (Table 5). However, patients with CAD did show a trend towards lower survival, but this was not statistically significant for cardiovascular death (HR 1.001, 95% CI 0.673–1.488, *p* = 0.996) and SCD (HR 0.802, 95% CI 0.395–1.627, *p* = 0.541).

**Table 4.** **Cox regression of cardiovascular morality in patients with HCM**.

Variables	Univariate analysis		Multivariate analysis	
	HR (95% CI)	*p* value	HR (95% CI)	*p* value
CAD	1.001 (0.673–1.488)	0.996		
Age	1.037 (1.019–1.055)	<0.001*	1.024 (1.006–1.041)	0.007*
Male	1.802 (1.208–2.687)	0.003*		
HOCM	0.809 (0.540–1.212)	0.304		
AF	1.778 (1.134–2.788)	0.012*		
Hyperlipemia	0.475 (0.278–0.812)	0.007*		
Heart rate	1.016 (1.006–1.027)	0.002*		
AST	1.006 (1.000–1.011)	0.031*		
TB	1.033 (1.009–1.058)	0.007*		
DB	1.141 (1.083–1.202)	<0.001*		
CR	1.002 (1.001–1.004)	<0.001*	1.003 (1.001–1.005)	0.001*
Log NT-proBNP	5.245 (3.499–7.861)	<0.001*	3.040 (1.843–5.014)	<0.001*
QT	0.994 (0.990–0.998)	0.002*	0.995 (0.991–0.999)	0.028*
IVS	1.021 (0.982–1.062)	0.300		
LA	1.037 (1.010–1.066)	0.007*		
LVEF	0.952 (0.932–0.971)	<0.001*	0.964 (0.943–0.985)	0.001*

**Notes: **“*” represents *p* < 0.05, indicating statistical significance. HCM, hypertrophic cardiomyopathy; HR, hazard ratio; 95% CI, 95% confidence interval; CAD, coronary artery disease; HOCM, hypertrophic obstructive cardiomyopathy; AF, atrial fibrillation; AST, aspartate aminotransferase; TB, total bilirubin; DB, direct bilirubin; CR, creatinine; NT-proBNP, N-terminal pro-B-type natriuretic peptide; IVS, interventricular septum; LA, left atrium; LVEF, left ventricular ejection fraction.

**Table 5.** **Cox regression of sudden cardiac death in patients with HCM**.

Variables	Univariate analysis		Multivariate analysis	
	HR (95% CI)	*p* value	HR (95% CI)	*p* value
CAD	0.802 (0.395–1.627)	0.541		
Age	0.987 (0.963–1.012)	0.313		
Male	0.975 (0.458–2.076)	0.948		
HOCM	1.271 (0.628–2.575)	0.505		
AST	1.009 (1.004–1.015)	<0.001*		
DB	1.115 (1.007–1.235)	0.036*		
CR	1.003 (1.001–1.004)	0.002*	1.003 (1.000–1.005)	0.028*
Log NT-proBNP	3.972 (1.884–8.374)	<0.001*		
IVS	1.064 (0.994–1.138)	0.073		
LA	1.063 (1.018–1.109)	0.005*		
LVEF	0.952 (0.918–0.986)	0.007*		

**Notes: **“*” represents *p* < 0.05, indicating statistical significance. HCM, hypertrophic cardiomyopathy; HR, hazard ratio; 95% CI, 95% confidence interval; CAD, coronary artery disease; HOCM, hypertrophic obstructive cardiomyopathy; AST, aspartate aminotransferase; DB, direct bilirubin; CR, creatinine; NT-proBNP, N-terminal pro-B-type natriuretic peptide; IVS, interventricular septum; LA, left atrium; LVEF, left ventricular ejection fraction.

### 3.4 Subgroup Analysis

**Fig. 4.**
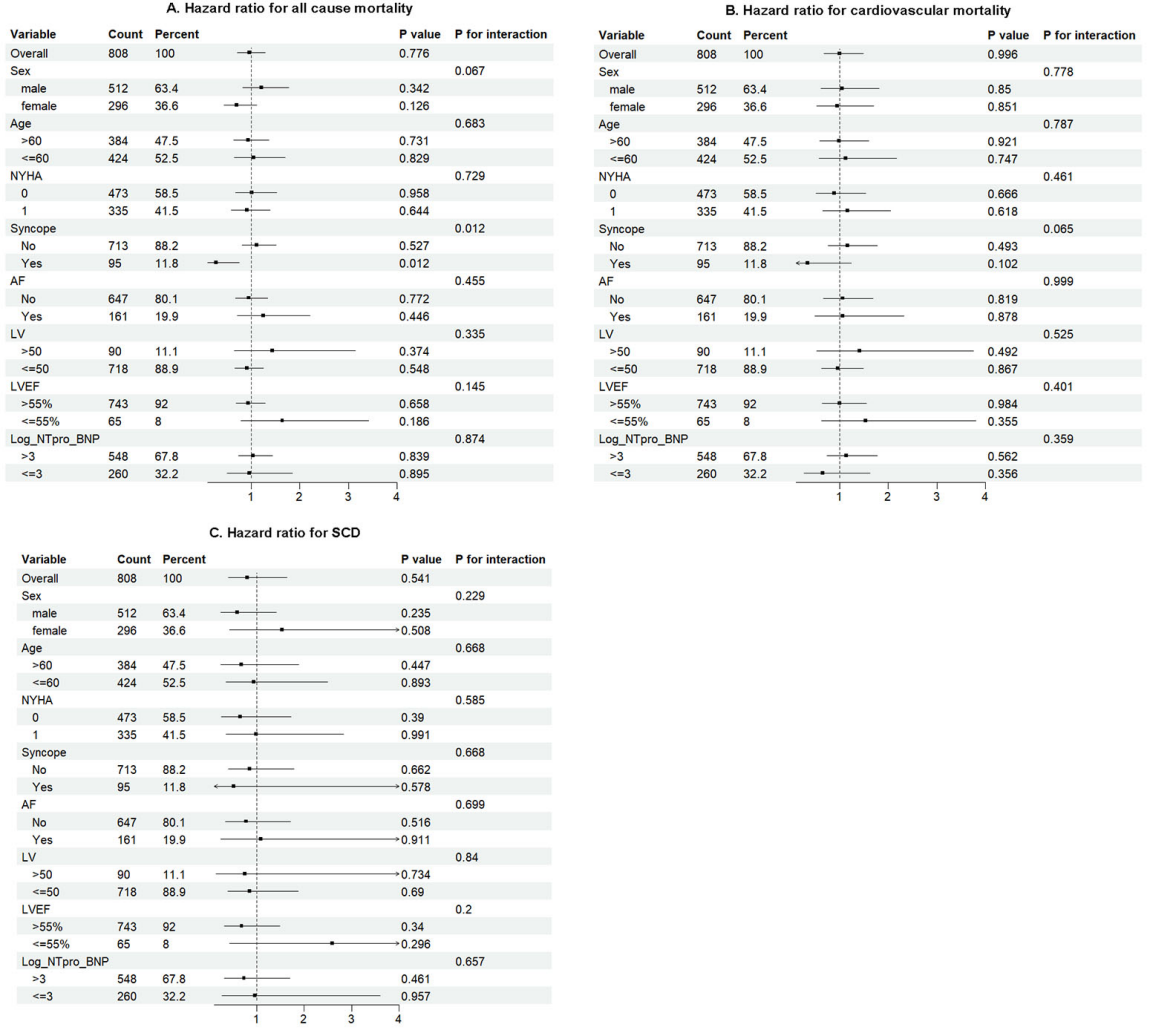
**Forest plots for all-cause mortality, cardiovascular mortality, and SCD in subgroup analyses**. (A) Forest plots for all-cause mortality. (B) Forest plots for cardiovascular mortality. (C) Forest plots for SCD. SCD, sudden cardiac death; NYHA, New York Heart Association; AF, atrial fibrillation; LV, left ventricular; LVEF, left ventricular ejection fraction; NT-proBNP, N-terminal pro-B-type natriuretic peptide.

To compare the effects of CAD in different subgroups, we conducted subgroup analyses using forest plots. Fig. 4 shows the effects of CAD on the different endpoint events for the different subgroups CAD was not significantly associated with all-cause mortality, cardiovascular death, or SCD in subgroups stratified by gender, age, New York Heart Association functional class, syncope, AF, left ventricular diameter, LVEF, or NT-proBNP in the matched cohort.

### 3.5 Sensitivity Analyses

By calculating the median of the non-missing values and assigning this median value to the missing values, the missing values are effectively imputed. Using univariate logistic regression, we carefully selected the indicators for PSM, ultimately identifying nine variables with *p* < 0.1 including age, hypertension, diabetes mellitus, tobacco use, family history of HCM, total bilirubin, direct bilirubin, high-density lipoprotein, and low-density lipoprotein. Then, the nine indicators were subjected to caliper matching, incorporated into a 1:1 PSM process, ultimately establishing a matched cohort of 406 HCM patients with CAD and 406 HCM patients without CAD. In the matched cohort, patients with and without CAD were not significantly different in terms of SCD (log-rank χ^2^ = 0.1, *p* = 0.948), cardiovascular death (log-rank χ^2^ = 0.1, *p* = 0.811), and all-cause mortality (log-rank χ^2^ = 0.5, *p* = 0.499).

The analysis of all-cause mortality from the multivariate Cox proportional hazards regression analysis reveals that age, creatinine, NT-proBNP, and LVEF were strong independent predictors of all-cause mortality. However, survival did not differ significantly between the CAD and non-CAD groups (HR 0.906, 95% CI 0.679–1.207, *p* = 0.499). Multivariate Cox proportional hazards regression analyses revealed that age, CR, NT-proBNP, and LVEF were independent predictors of cardiovascular death. In addition, after adjusting for other related variables, only CR was an independent predictor of SCD. However, there is no statistical significance in cardiovascular death (HR 1.049, 95% CI 0.711–1.547, *p* = 0.811) and SCD (HR 1.023, 95% CI 0.516–2.027, *p* = 0.948) among patients with coronary heart disease. Both imputation methods indicated no statistically significant differences in all-cause mortality, cardiovascular mortality, and sudden death between patients with CAD and HCM.

## 4. Discussion

In this cohort study, we evaluated the association of CAD with SCD, cardiovascular death, and all-cause mortality in patients with HCM. The Kaplan–Meier survival curve did not show any statistically significant differences in any of the endpoint events between the CAD and non-CAD groups, suggesting that CAD is not a predictor of SCD, cardiovascular death, or all-cause mortality in patients with HCM.

HCM is a genetic heart disease that affects people globally [14]. However, several comorbidities may be more hazardous to survival than long-term HCM. Around 10% of patients with HCM demonstrate a reduction in regional blood flow owing to concomitant epicardial CAD, which is a sign of a poor prognosis in HCM [15]. According to Sorajja *et al*. [10], individuals with severe CAD are at an increased risk of death. Moreover, according to Shin *et al*. [16], in patients with HCM, CAD is an independent risk factor for cardiovascular events. Myocardial ischemia caused by microvascular dysfunction is an acknowledged pathophysiological factor in HCM [17]. In patients with HCM, there are many complex putative mechanisms that may lead to ischemia, such as myocardial bridging, demand arising from the hypertrophied myocardium, and intramural CAD [1, 17, 18]. Left ventricular hypertrophy and stiffness lead to an increase in intraventricular pressure, which may further impair myocardial perfusion, resulting in myocardial ischemia during exercise [19]. Hypertrophic myocardium found on the pathology of microvascular remodeling, small intramural coronary artery perivascular fibrosis lesions of luminal stenosis, the mechanism for coronary blood flow reserve reserves to reduce [20]. Although concomitant atherosclerotic disease and its related morbidities can aggravate the anatomical anomalies and the innate endothelium impairment in HCM, some studies have proven that ischemia unrelated to CAD is a predictor of endpoint events in patients with HCM [21, 22]. This ischemia is caused by insufficient myocardial perfusion, microvascular dysfunction, and reduced coronary flow reserve [22, 23, 24]. In the present study, there did not appear to be any significant patterns in survival between the CAD and non-CAD groups, but the CAD group showed a higher mortality rate than the non-CAD group.

A few studies have reported the existence of CAD in patients with HCM, but only a couple of small investigations have reported their long-term results. Therefore, it is uncertain how CAD affects the prognosis of patients with HCM [9, 10]. Myocardial ischemia is the trigger for some fatal consequences in patients with HCM, including systolic dysfunction, ventricular arrhythmia, progressive left ventricular remodeling, and even sudden death [10, 25, 26]. Evidence from earlier investigations has demonstrated that hemoperfusion abnormalities are present in more than 50% of patients with HCM, and a common adverse effect of HCM is myocardial ischemia [5]. In the present study, 22.3% of the patients with HCM were diagnosed with CAD, which is similar to the rate of 19%–26% reported previously [10, 25]. Patients with microvascular dysfunction resulting from the increased high myocardial oxygen demand and myocardial mass demonstrated an increased prevalence of negative outcomes [27]. Health care has improved in China over the years, the medical quality is better, more patients enjoy high quality health care, health education is also very successful. There has also been an increase in HCM- related gene screening in recent years, which can improve the prognosis of patients with HCM to be better than ever before. In this retrospective study, the mortality rate of patients with HCM and CAD was numerically higher than that of patients without CAD. However, the mortality rate was not significantly different between the two groups. Patients with CAD tend to pay more attention to physical exercise in their daily lives and reduce behaviors that may exacerbate their conditions. Patients with HCM and CAD are more cautious about taking medications, and research has shown that with effective treatment, patients with CAD are likely to have a better prognosis [28, 29].

This study has some limitations. First, because this was a retrospective real-world clinical investigation, some inherent biases may be present. We were limited to using the patients’ existing data. In addition, similar to other hospital-based cohorts, a selected group of patients who were assigned for treatment were the study population under consideration. Second, given that this is a multi-center cohort study, regional heterogeneity is inevitable. Fortunately, the baseline characteristics of the two subgroups were comparable, which indicated that patient selection bias was acceptable. Third, although Sorajja *et al*. [10] found that CAD tends to be positively correlated with mortality, our multivariate analysis revealed that there was no statistically significant difference between the CAD and non-CAD groups. We think that the limited sample size may have contributed to this, and a larger sample size would have improved our results. Finally, the Fu Wai Hospital of the Chinese Academy of Medical Sciences is one of the best cardiovascular hospitals in China, attracting patients from all over the country for treatment, resulting in significantly more patients with cardiomyopathy at this center than at others.

## 5. Conclusions

In this study, it was observed that there was no statistically significant disparity in mortality rates between patients diagnosed with HCM who concurrently had CAD and those who did not exhibit CAD. This finding underscores the notion that the presence of CAD did not exert a notable influence on the incidence of SCD, cardiovascular death, or all-cause mortality, thereby emphasizing the complexity and multifaceted nature of mortality risk factors in HCM patients.

## Availability of Data and Materials

The datasets generated for this study are available on request to the corresponding author.
